# Preparation and Characterization of Liquisolid Compacts for Improved Dissolution of Telmisartan

**DOI:** 10.1155/2014/692793

**Published:** 2014-10-12

**Authors:** Naveen Chella, Nataraj Narra, Tadikonda Rama Rao

**Affiliations:** ^1^National Institute of Pharmaceutical Education and Research, Hyderabad, Andhra Pradesh 500037, India; ^2^Department of Pharmacy, Acharya Nagarjuna University, Guntur, Andhra Pradesh 522 510, India; ^3^Avanthi Institute of Pharmaceutical Sciences, Hyderabad, Andhra Pradesh 501512, India

## Abstract

The objective of the present work was to obtain pH independent and improved dissolution profile for a poorly soluble drug, telmisartan using liquisolid compacts. Liquisolid compacts were prepared using Transcutol HP as vehicle, Avicel PH102 as carrier, and Aerosil 200 as a coating material. The formulations were evaluated for drug excipient interactions, change in crystallinity of drug, flow properties, and general quality control tests of tablets using Fourier transform infrared (FTIR) spectroscopy, differential scanning calorimetry (DSC), X-ray diffraction (XRD), angle of repose, and various pharmacopoeial tests. *In vitro* dissolution studies were performed at three pH conditions (1.2, 4.5 and 7.4). Stability studies were performed at 40°C and 75% RH for three months. The formulation was found to comply with Indian pharmacopoeial limits for tablets. FTIR studies confirmed no interaction between drug and excipients. XRD and DSC studies indicate change/reduction in crystallinity of drug. Dissolution media were selected based on the solubility studies. The optimized formulation showed pH independent release profile with significant improvement (*P* < 0.005) in dissolution compared to plain drug and conventional marketed formulation. No significant difference was seen in the tablet properties, and drug release profile after storage for 3 months.

## 1. Introduction

Recent developments in combinatorial chemistry and high-throughput screening used in drug discovery resulted in increased number of drugs with poor aqueous solubility. Approximately 90% of the new chemical entities (NCEs) are considered poorly soluble with either high or low permeability (Biopharmaceutics Classification System (BCS) class II and IV) [1]. BCS class II drugs have high absorption number and low dissolution number. The absorption/bioavailability is limited by dissolution rate. These drugs exhibit varying bioavailability and small increment in dissolution may result in substantial improvement in bioavailability. Hence, dissolution enhancement is the key factor in formulating BCS class II drugs [2].

Telmisartan, 4′-[(1,4′-dimethyl-2′-propyl[2,6′-bi-1*H*-benzimidazol]-1′-yl)methyl][1,1′-biphenyl]-2-carboxylic acid, is a potent and selective AT_1_ receptor antagonist used in the treatment of essential hypertension [3]. Telmisartan is a BCS class II drug (aqueous solubility is 0.09 *μ*g/mL) [4] with pH-dependent solubility (practically insoluble in the range of pH 3–9) and is highly hydrophobic in nature (log *P* = 3.2; n-octanol/buffer at pH 7.4) [5]. Preclinical studies on telmisartan pharmacokinetics also report significant variation in the pharmacokinetic parameters (*C*_max⁡_ and *t*_max⁡_) under fed and fasted conditions [6]. The poor aqueous solubility of drug is associated with slow drug dissolution and slow/erratic absorption leading eventually to inadequate and low oral bioavailability (43%) [7].

Similarly, to date, all the drug delivery approaches reported including self-emulsifying drug delivery (SEDDS) [8], solid dispersion [9], and complexation with cyclodextrin [10] were aimed at improving the dissolution or use of alkalizers [4] to modulate the microenvironmental pH so that dissolution can be improved. However, there are some practical problems with the abovementioned techniques, like stability of drug with the use of pH modifiers and formulation stability in case of solid dispersions, toxicity due to high amounts of surfactants used in the formulation of SEDDS [11], toxicity associated with the use of cyclodextrins [12], and scale-up feasibility. Hence, there is a need of delivery system that is easy to manufacture with scale-up feasibility and contains excipients which are safe.

Liquisolid compaction (LSC) based on powder solution technology shows promising potential in improving the dissolution rate of poorly soluble drugs like telmisartan. LSC technology not only enhances the drug dissolution but can be commercially viable and has industrial scale-up feasibility due to low cost and ease of handling. LSC technology is used to convert liquid medications into solid systems. The liquid medication, that is, drug dissolved or dispersed in a nonvolatile solvent is converted into a dry, nonadherent, free flowing, and compressible powder blend by mixing with selected carriers and coating materials. The compounds with high porous surface and high absorption properties such as cellulose derivatives, starch, and lactose can be used as carrier and Aerosil can be used as coat material. Spireas and Bolton developed a mathematical model to calculate the amount of carrier and coating material required to produce an acceptable flow and compressibility [13].

The success of liquisolid system with an acceptable flow rate and compressibility depends on liquid load factor (*L*_*f*_) and excipient ratio (*R*). The liquid load factor (*L*_*f*_) is a characteristic of amount of vehicle used in the formulation that is defined as the weight ratio of the liquid medication (*W*) and carrier. The excipient ratio (*R*) of a powder is defined as the ratio between the weights of carrier (*Q*) and coating material (*q*) present in the formulation. Hence, the powder excipients ratio and liquid load factor of the formulations are related as follows [14]:

(1) $$\begin{array}{c} L_{f}=\varphi +\varphi \left(\frac{1}{R}\right), \end{array}$$

where **φ** and *φ* are flowable liquid-retention potential of carrier and coat material, respectively.

To date, liquisolid systems were formulated using drug solution [15–18]; however the efficacy of this system to improve the dissolution rate with drug suspension has not been thoroughly investigated. The secondary objective of the study is to formulate liquisolid system with drug suspension and to evaluate its efficacy.

## 2. Materials and Methods

### 2.1. Materials

Telmisartan was kindly gifted by Aurobindo Pharmaceuticals (Hyderabad, India). Transcutol HP is a kind gift from Gattefosse India Pvt. Ltd. (Mumbai, India). Tween 20, Tween 80, polyethylene glycol (PEG-200 and PEG 600), propylene glycol (PG), sodium hydroxide, sodium lauryl sulphate (SLS), and potassium dihydrogen orthophosphate were purchased from S D Fine-Chem Ltd. (Mumbai, India). Avicel PH102, Aerosil 200, lactose, dicalcium phosphate (DCP), and croscarmellose sodium were purchased from Nehal Traders (Hyderabad, India). All other chemicals, reagents, and solutions used were of analytical grade. Marketed product Telma 20 mg for immediate release (Glenmark Pharmaceutical Ltd., Himachal Pradesh, India) was procured from local pharmacy.

### 2.2. Solubility Studies

The solubility studies of the drug were carried out in different nonvolatile solvents and in different media at different pH, PEG 200, PEG 600, Transcutol HP, Tween 20, Tween 80, and propylene glycol, and in 0.1 N HCl (pH 1.2), phosphate buffer (pH 7.4), and saline phosphate buffer (PBS) with 0.1% and 0.5% sodium lauryl sulphate (SLS). The solubility determination was carried out by shake flask method [19]. An excess amount of drug was added to the vials containing selected vehicles. The vials were sealed and the mixture was vortexed using a vortex mixer for 10 min in order to facilitate proper mixing of drug with the vehicles and subjected to shaking on the incubator shaker (JEIOTECH, Korea) for 48 h at 25 ± 1°C. After this period the solutions were centrifuged and supernatant was separated. The supernatant was filtered through a 0.45 *μ*m Millipore filter and analysed for drug content by UV spectrophotometer (JASCO V-650, Japan). The determinations were carried out thrice for each sample and its mean along with standard deviation was reported.

### 2.3. Preformulation Studies

*Flow Properties of Liquisolid Powders*. Flow properties were determined on prepared liquisolid powder before compression of tablets. Angle of repose was measured by fixed funnel and free standing cone method [20]. A funnel was fixed at a given height *H*, above a graph paper placed on a flat horizontal surface. The powders were carefully poured through the funnel until the apex of the conical pile just touches the tip of the funnel. The mean radius *R*, of the base of the conical pile, was determined and the tangent of the angle of repose was given by Tan *α* = *H*/*R*, where *α* is angle of repose.

### 2.4. Preparation of Liquisolid Compacts

The required amount of drug (20 mg) was weighed and transferred to a mortar and dispersed in different nonvolatile liquids (Transcutol HP, propylene glycol, PEG 200, PEG 600, Tween 20, and Tween 80) to get 20% w/w concentration. From the literature studies it was found that excipient ratio at 20 will produce tablets with sufficient hardness and better dissolution profiles. Hence, the excipient ratio (*R*) was kept at constant value 20. The calculated amount of carrier and coating material was added to the dispersion and blended in a porcelain mortar avoiding excessive trituration and particle size reduction as described by Spireas et al., [21]. To this, 40 mg of croscarmellose sodium was added and mixed thoroughly. The final mixture was compressed into tablets manually using multistation rotary punching machine (Rimek Minipress I, Karnavati Engineering Pvt. Ltd., Gujarat, India). The compression force was adjusted depending on the weight of tablet and ingredients in the formulation. Forty tablets were prepared in a batch for each formulation. Formulation parameters are summarized in Table 1.

Physical mixture was prepared by mixing drug, Avicel, and Aerosil in same amount as used in the formulation in a mortar. The physical mixture did not contain any vehicle.

### 2.5. Evaluation of Liquisolid Tablets

The prepared liquisolid tablets were evaluated for weight variation, content uniformity, hardness, friability, and disintegration time. Hardness was determined by Pfizer hardness tester and friability by digital tablet friability tester. The disintegration time was measured using USP disintegration tester (Electrolab India PVT. LTD., Mumbai, India). Flow properties were measured in terms of angle of repose. All the studies were done in triplicate.

#### 2.5.1. FTIR Spectroscopy

The FTIR spectra of drug, Avicel PH102, Aerosil, physical mixture, and LSC formulation were recorded on Perkin Elmer spectrophotometer (PerkinElmer Inc., MA, US) using KBr pellet from 4000 cm^−1^ to 400 cm^−1^ range. The pellets were prepared by mixing 5 mg of sample with 100 mg potassium bromide and compacted under vacuum at a pressure of about 12,000 psi for 3 minutes.

#### 2.5.2. X-Ray Powder Diffraction (XRD)

The XRD patterns of drug, physical mixture, and optimized LSC formulation were recorded at room temperature on Simens D5000 X-ray diffractometer using Ni-filtered Cu K*α* radiation (wavelength 1.540 Å). The data was recorded over a scanning 2*θ* range of 2° to 50°.

#### 2.5.3. Differential Scanning Calorimetry (DSC)

DSC studies were performed using a Mettler DSC 1 (Mettler Toledo, Germany). The instrument was calibrated with an indium standard. Accurately weighed samples (5–10 mg) were placed in closed, pierced, flat bottom aluminium pans. DSC scans were recorded at a constant heating rate of 10°C/min from 30 to 350°C. Nitrogen gas was pumped at a flow rate of 80 mL/min [22]. The melting point, peak maxima, appearance of any new peak, and change in peak shape were noted.

### 2.6. *In Vitro* Dissolution Studies

The USP paddle method was used for all* in vitro* dissolution studies. Dissolution studies were performed in 900 mL of 0.1 N HCl (pH 1.2), acetate buffer with 0.5% SLS (pH 4.5), and saline phosphate buffer with 0.5% SLS (pH 7.4) maintained at 37.5 ± 0.5°C. The rate of stirring was 75 ± 1 rpm. At appropriate intervals (5, 10, 15, 30, 45, 60, 90, and 120 min), 5 mL of samples was taken and filtered through a 0.45 micron filter. The samples were analyzed at 298 nm by UV-visible spectrophotometer. The mean of three determinations was used to calculate the drug release from each formulation.

### 2.7. Mathematical Modelling of Drug Release

For the comparison of dissolution data, percentage of drug dissolved at 15 min (*Q*_15 min⁡_), 30 min (*Q*_30 min⁡_), mean dissolution time (MDT), and dissolution efficiency (DE_30_) at 30 min were calculated. MDT defined as arithmetic mean value of the given dissolution profile was calculated as follows [23]:

(2) $$\begin{array}{c} \text{MDT}=\frac{\sum_{i=1}^{n}t\Delta M_{i}}{\sum_{i=1}^{n}\Delta M_{i}}, \end{array}$$

where “*i*” is the sample number, *n* is the number of dissolution sample times, *t* is the time at the midpoint between *t* and *t* − 1 (calculated with (*t* + *t* − 1)/2), and Δ *M*_*i*_ is the additional amount of drug dissolved between *t* and *t* − 1.

Dissolution efficiency DE is given by formula,

(3) $$\begin{array}{c} \text{DE}=\frac{\int_{0}^{t}Ydt}{\int_{0}^{t}Y_{100}t}, \end{array}$$

where “*Y*” is the percent of drug released as a function of time, *t* is the total time of drug release, and “*Y*_100_” is 100% drug release [24].

Similarity factor (*f*_2_) is calculated by using equation proposed by Moore and Flanner which is as follows [25]:

(4) $$\begin{array}{c} f_{2}=50\log\left\{{\left[1+\frac{1}{n}\overset{n}{\underset{t=1}{\sum }}W_{t}{\left(R_{t}-T_{t}\right)}^{2}\right]}^{-0.5}\times 100\right\}, \end{array}$$

where “*R*_*t*_” and “*T*_*t*_” are the cumulative percentage dissolved at each of the selected *n* time points of the reference and test product, respectively. The factor *f*_2_ measures the closeness between the two profiles. FDA has set a standard of *f*_2_ value 50–100 to indicate similarity between two dissolution profiles. The two profiles are identical, if *f*_2_ = 100 and *f*_2_ value less than 50 indicates difference in profiles.

### 2.8. Stability Studies

Stability of optimised formulation was carried out according to the ICH guidelines. The optimized formulation was stored at 40°C and 75% RH for 3 months and effect on release profile and the crushing strength were compared with those of freshly prepared tablets.

### 2.9. Statistical Analysis

The difference in the dissolution rate of drugs from different formulations, plain drug, and marketed tablet was evaluated by one way ANOVA or paired* t*-test at a level of *P* = 0.05.

## 3. Results and Discussion

The drugs with poor aqueous solubility, high hydrophobic nature (log *P* > 3), and pH dependent solubility make it challenging to develop a dosage form with desired dissolution rate* in vivo* performance [7]. Telmisartan is an example of such category. Hence, the present work was designed to investigate the effectiveness of liquisolid technology to obtain improved dissolution rate and pH independent drug release.

### 3.1. Solubility Studies

Solubility studies in different media at different pH were conducted to find a suitable dissolution media that provides sink condition. The solubility of telmisartan in different nonvolatile solvents and at different pH was presented in Table 2. Different researchers reported different values for aqueous solubility ranging from 0.09 to 35 *μ*g/mL [4, 26, 27]. In our laboratory conditions, telmisartan showed poor aqueous solubility (22.47 *μ*g/mL) which is in accordance with literature cited. The solubility also changed with change in pH conditions (Table 2). The solubility studies in different dissolution media indicated that the dose of telmisartan (20 mg) taken was not soluble in 900 mL of acetate and phosphate buffer under normal conditions. This low solubility makes it difficult to maintain the sink condition in dissolution media. Hence, SLS was used as solubilizer (0.5% w/v) to enhance the solubility and to maintain sink conditions in the respective dissolution media. Solubility of drug in nonvolatile solvents was conducted to determine the approximate volume of solvent required to disperse the drug.

To avoid the interference from the solvent and to confirm the absorbance showed was due to drug only all the vehicles used in the formulation were diluted with respective dissolution media and scanned over the range of 200–400 nm. The vehicles showed less absorbance (less than 0.05) at very high concentrations (1% w/v; well above the concentrations used in the formulation) which are negligible.

### 3.2. Preformulation Studies

#### 3.2.1. Flow Properties

The angle of repose is characteristic of the internal frictional forces of the particles. Angle of repose will be high if the particles are cohesive. Values for angle of repose ≤30° indicate free flow and angles ≥40° indicate poor flow [28]. LSC formulations (F2–F6) showed angle greater than 30° indicating poor flow.

### 3.3. Evaluation of LSC Formulation

Different evaluation parameters like hardness, flow properties, friability, and disintegration time were reported in Table 3. The fundamental requirement for all dosage forms is to maintain constant dose of drug between each unit in a batch. Formulations F1–F4 complied with content uniformity as per Indian pharmacopoeia with 96.43%, 88.02%, 86.28%, and 87.89%, respectively. Formulations F5 and F6 showed 83.25% and 81.63% content uniformity, respectively, which does not comply with IP limits (85–110%) [29]. This may be due to insufficient quantity of the liquid to wet the amount of drug taken which in turn failed to distribute the drug with carrier and coating material during mixing process. All the liquisolid tablets (F1–F6) had acceptable friability as none of the tested formulae had percentage loss more than 1% [30] and no tablet showed cracking, splitting, or broken pieces. All the formulations showed weight variations within limits (<±5%) as per Indian pharmacopoeia and passed the test.

Generally the tablets should be sufficiently hard to resist breaking during normal handling and yet soft enough to disintegrate properly after swallowing. Formulations F1, F2, and F6 showed acceptable hardness (3-4 kg/cm^2^) whereas F4 and F5 showed hardness less than 2 kg/cm^2^ [30]. Formulation F1 disintegrates within 1.2 min; other formulations took around 3–5 minutes (Table 3) to disintegrate totally. The longer disintegration time may retard the release of drug from the dosage form. Hence those were not included for further studies. Based on the flow properties, hardness, and disintegration time formulation F1 was selected as optimized formulation and further characterization was performed.

**Figure 1:**
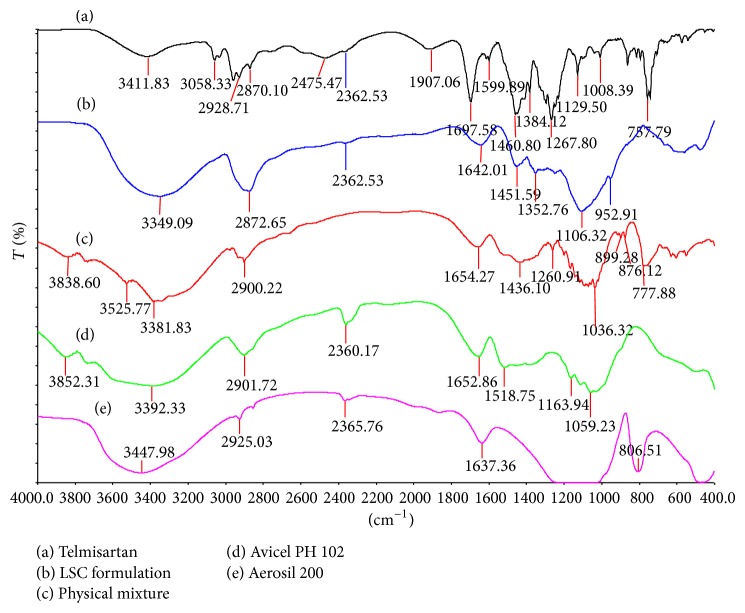
FTIR spectra of telmisartan plain drug, excipients, physical mixture, and final optimized formulation (F1) (refer to Table 1 for formulation composition).

FTIR analyses provide information on physicochemical properties of substances with respect to compatibility [31]. FTIR and spectra of telmisartan, Aerosil 200, Avicel PH102, and physical mixture of drug, carrier/coat material, and optimised formulation (F1) are shown in Figure 1. IR spectrum (Figure 1) of telmisartan exhibits characteristic peaks at 3446 cm^−1^ (N–H stretch), 3063 cm^−1^ (aromatic C–H stretch), 2957 cm^−1^ (aliphatic C–H stretch), 1697 cm^−1^ (carbonyl group), and 1599 cm^−1^ (aromatic C=C bend and stretch) and the peak at 1458 cm^−1^ indicates the presence of C=C aromatic group. Appearance of all these peaks and absence of any new peaks in the physical mixture and liquisolid formulation indicate no chemical interaction between the drug and excipients.

**Figure 2:**
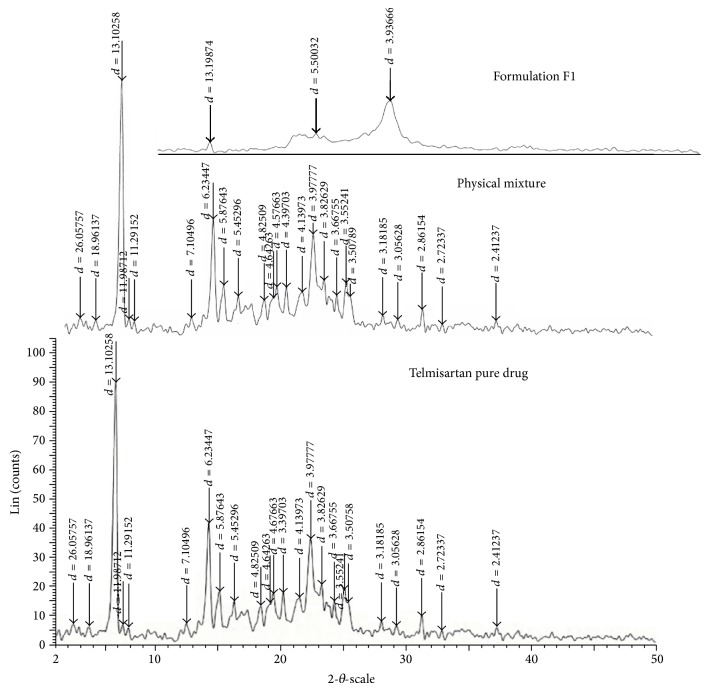
XRD spectra of plain drug, physical mixture, and optimized formulation (F1) (refer to Table 1 for composition of formulation).

The powder XRD technique is to fingerprint a specific solid form of the crystalline API. Any changes during the product development or formulation can be identified using this technique. The crystalline form changes can have a large impact on the bioavailability of the molecule due to the changes in solubility and thereby its dissolution [32]. The XRD patterns of telmisartan (Figure 2) show sharp distinct peaks notably at 2*θ* diffraction angles of 6°, 14°, 15°, and 22° indicating telmisartan was in crystalline state. The appearance of peaks at same diffraction angles in the XRD of physical mixture indicates crystalline structure remained unchanged. Similarly, the disappearance or decrease in intensity of the peaks at same diffraction angles in optimized formulation indicates that telmisartan may have undergone solid state transition from crystalline to amorphous form or crystallinity was reduced.

**Figure 3:**
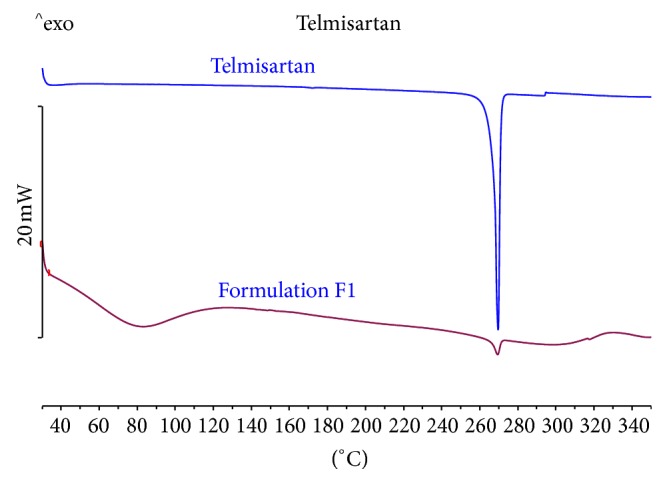
DSC thermogram of plain drug and optimized formulation (F1) (refer to Table 1 for formulation composition).

The DSC thermogram (Figure 3) of telmisartan showed sharp endothermic peaks with onset temperature of 266.45°C and peak temperature 268.74°C corresponding to its melting point. The presence of same peak in the final formulation indicates that there was no interaction between drug and excipients during the formulation process. The decrease in the enthalpy (Δ*H*) indicates reduction in crystallinity or partial amorphization [33] of the drug which is in confirmation of XRD results.

### 3.4. *In Vitro* Dissolution Studies

**Figure 4:**
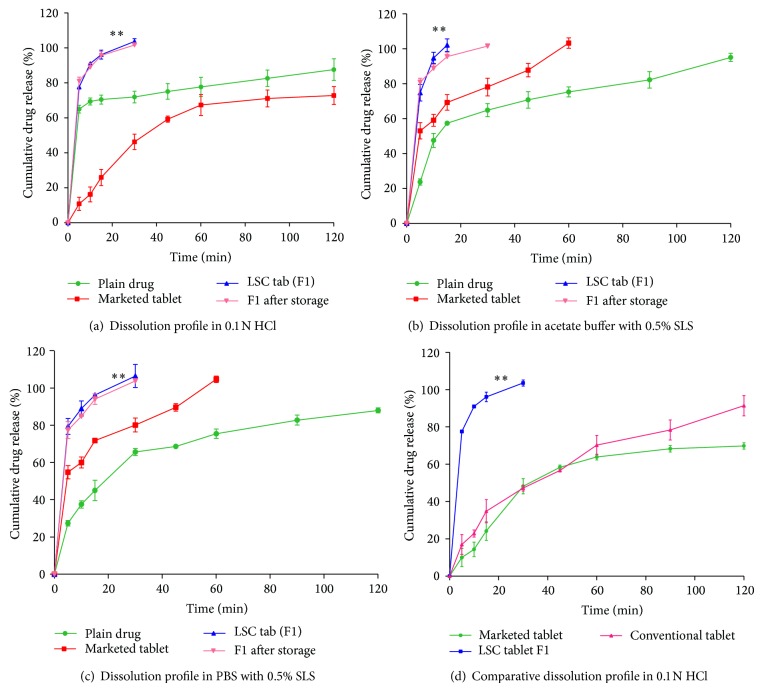
Dissolution profile of telmisartan plain drug, marketed tablet, optimized formulation (F1), and F1 after storage in different media (refer to Table 1 for formulation composition).

The solubility decreases with increasing pH. Hence, buffers with higher pH (acetate buffer pH 4.5 and phosphate buffer pH 7.4) were selected to perform the dissolution studies which act as discriminating media. To maintain sink conditions, the dissolution studies were performed in respective media with the aid of solubilizer like SLS in 0.5% w/v concentrations.  Figure 4 shows cumulative (%) drug released from plain drug, marketed product, and optimised formulation (F1) in 0.1 N HCl, ABS, and PBS with 0.5% SLS at different time intervals. MDT, *Q*_15 min⁡_ , *Q*_30 min⁡_, and DE at 30 min were reported in Table 4. The dissolution profile (Figure 4) showed complete drug release in 30 min from LSC formulation compared to 46.3% from marketed tablet and 71.82% from plain drug in 0.1 N HCl. The reduced dissolution of marketed product would be due to slow disintegration of the tablet in the dissolution media. In ABS with 0.5% SLS complete drug release was observed in 15 min with formulation F1 whereas less than 60% of drug was released from plain drug and marketed formulation. The optimised telmisartan LSC formulation, F1, showed complete drug release in 30 min compared to 82.90% from marketed tablet and 65.62% of drug release from plain drug in phosphate buffer containing 0.5% SLS. The results correlate to telmisartan low solubility at higher pH conditions.

### 3.5. Dissolution Data Treatment

The improvement in the dissolution was further confirmed by comparing the various dissolution parameters calculated from dissolution data of plain drug, marketed tablet, and LSC formulation F1. In all three dissolution media (*Q*_15 min⁡_) values were around 95% from the LSC tablets which were comparatively higher and significant (*P* < 0.05) compared to plain drug and conventional marketed tablet. The increase in dissolution was further confirmed from (*Q*_30 min⁡_) values that showed 100% for formulations in all the three dissolution media compared to 71.82%, 64.85%, and 65.62% for plain drug and 46.30%, 78.15%, and 82.90% for marketed product in 0.1 N HCl, acetate buffer, and PBS with 0.5% SLS, respectively.

The dissolution results were further supported by significantly higher (*P* < 0.05) dissolution efficiency values of LSC formulation compared to plain drug and marketed tablet at 30 min. DE_30 min⁡_ was increased from 1.96% for marketed product and 9.44% for plain drug to 100% for LSC formulation in 0.1 N HCl. Similarly in acetate buffer and PBS with 0.5% SLS also LSC formulation showed significantly higher values compared to plain drug and marketed product. The decrease in mean dissolution time (MDT) values indicates the faster release of drug from formulation F1 compared to plain drug and marketed tablet in all the three dissolution media.

This increase in the dissolution with LSC formulation was attributed to the increased wettability and surface availability of drug to the dissolving medium as drug is molecularly dispersed within a water miscible solvent like Transcutol HP. This release can be further explained with the help of Noyes-Whitney equation:

(5) $$\begin{array}{c} \frac{dC}{dt}=\frac{\text{DS}\left(C_{s}-C\right)}{h}. \end{array}$$

From the above equation the dissolution rate *dC*/*dt* is directly proportional to the surface area of drug available for dissolution medium (*S*) and the drug concentration gradient (*C*_*s*_ − *C*). An increase in surface area increases the dissolution. However, in case of plain drug and marketed tablet the surface area exposure of drug particle to dissolution was limited due to the hydrophobicity of the drug particles while in case of LSC the drug is dispersed at molecular level leading to apparent increase in surface area available for dissolution. Further, the increase in dissolution may also be due to reduction in crystallinity of the drug in LSC which was confirmed from XRD and DSC studies. Such higher drug dissolution rates displayed by liquisolid compacts may also imply enhanced oral bioavailability.

The release of telmisartan from F1 was also compared with that of directly compressed tablet (Figure 4) prepared in the similar manner without nonvolatile liquid to study the contribution of nonvolatile solvents to the drug dissolution. The release from directly compressed tablet was only 44% in 30 min compared to complete drug release from liquisolid formulation. The difference in dissolution was found to be significant (*P* < 0.05) using paired* t*-test analysis. This clearly indicates the improvement in the dissolution of telmisartan was due to presence of drug in nonvolatile solvent in liquisolid formulation.

Similarity factor (*f*_2_) value of 65 for telmisartan in acetate buffer and PBS dissolution profiles with reference to dissolution in 0.1 N HCL indicates the drug showed improved dissolution in all the three media (pH 1.2, 4.5, and 7.4) which were identical [34]. Hence, telmisartan was found to show pH independent release.

### 3.6. Stability Studies

No significant difference (*P* > 0.05) was found in the hardness and dissolution rate between the stored formulations when compared with freshly prepared formulation in all the media.

## 4. Conclusion

The present study concludes that the liquisolid compaction was found to be a promising technique for improving the dissolution of poorly soluble drug like telmisartan. The LSC formulated with Transcutol HP was found to be a better product with pH independent and improved dissolution profile and acceptable tableting properties.* In vitro* dissolution studies showed dissolution improvement from LSC tablets when compared to plain drug and marketed tablet. The dissolution data treatment using different parameters further confirmed the improvement in dissolution. Similarity factor indicated dissolution profile obtained at different pH was identical confirming pH independent release of telmisartan. XRD and DSC studies indicated reduction in crystallinity, a factor contributing to dissolution rate improvement of the drug, and IR spectra indicate there were no interactions between drug and excipients. The stability studies showed that the dissolution of liquisolid formulation was not affected by ageing significantly.

## Figures and Tables

**Table 1:** Formulation details of telmisartan liquisolid tablets.

Formulation∗	Vehicle	*L*_*f*_^+^	Avicel PH 102 (mg)	Aerosil 200 (mg)	Weight of tablet (mg)^#^
F1	Transcutol HP	0.247	340	17	417
F2	Propylene glycol	0.224	375	18.75	453.75
F3	Polyethylene glycol 200	0.107	400	20	480
F4	Polyethylene glycol 600	0.101	500	25	585
F5	Tween 20	0.158	695	35	790
F6	Tween 80	0.139	610	30	700

^*^Fixed powder excipient ratio (*R* = *Q*/*q*) equal to 20 was used.

^+^Liquid load factor is defined as *L*_*f*_ = *W*/*Q*.

^#^Final tablet weight includes 40 mg of disintegrant and 1% of lubricant.

**Table 2:** Solubility of telmisartan at different solvents and media (mean ± SD; *n* = 3).

Media	Solubility (mg/mL)	Nonvolatile solvents	Solubility (mg/mL)
Distilled water (pH 6.8)	0.022 ± 0.02	Tween 20	4.2 ± 0.61
0.1 N HCl (pH 1.2)	0.549 ± 0.04	Tween 80	5.5 ± 0.45
Acetate buffer (pH 4.5)	0.033 ± 0.01	PEG 200	9.2 ± 0.86
Acetate buffer with 0.5% SLS (pH 4.5)	0.233 ± 0.09	PEG 600	8.8 ± 0.22
Saline phosphate buffer (PBS) (pH 7.4)	0.013 ± 0.01	Propylene glycol	3.2 ± 0.9
PBS with 0.5% SLS (pH 7.4)	0.108 ± 0.04	Transcutol P	6.1 ± 0.42

**Table 3:** Evaluation parameters of LSC formulations (mean ± SD; *n* = 3).

Formulation∗	Friability (%)	Hardness (kg/cm^2^)	Disintegration tsime (min)	Angle of repose
F1	0.54 ± 0.03	4	1.2 ± 0.08	29.64 ± 1.17
F2	0.68 ± 0.16	3	3.12 ± 0.13	34.64 ± 0.89
F3	0.63 ± 0.21	3	5.28 ± 0.05	45.23 ± 1.88
F4	0.59 ± 0.15	2	5.14 ± 0.04	45.03 ± 1.66
F5	0.66 ± 0.22	2	4.28 ± 0.11	35.06 ± 0.98
F6	0.64 ± 0.05	3	4.19 ± 0.09	39.24 ± 2.24
F1 after storage	0.52 ± 0.15	4	1.4 ± 0.12	28.92 ± 2.12

^*^Refer Table 1 for formulation composition.

**Table 4:** Dissolution parameters of plain drug, marketed product, and LSC formulation.

Dissolution parameter	0.1 N HCl			ABS (0.5% SLS)			PBS (0.5% SLS)		
	PD	MT	F1	PD	MT	F1	PD	MT	F1
*Q*_15 min_ (%)	70.41 ± 2.54	25.93 ± 4.60	96.08 ± 2.53	57.35 ± 1.18	69.28 ± 4.46	102.12 ± 3.54	45.01 ± 7.73	75.33 ± 2.02	96.31 ± 1.24
*Q*_30 min_ (%)	71.82 ± 3.32	46.30 ± 4.38	103.6 ± 1.61	64.85 ± 3.72	78.15 ± 5.07	102.12 ± 3.54	65.62 ± 2.66	82.90 ± 2.89	106.5 ± 6.2
DE_30_ (%)	9.44 ± 0.44	1.96 ± 0.74	100 ± 1.38	9.97 ± 0.12	40.61 ± 0.83	100 ± 1.14	6.93 ± 0.69	25.57 ± 0.02	100 ± 1.66
MDT (min)	32.02 ± 0.20	62.97 ± 2.86	4.78 ± 0.17	22.03 ± 1.82	13.65 ± 1.62	3.35 ± 0.6	38.13 ± 2.36	72.51 ± 4.44	4.36 ± 1.86

PD: plain drug.

MT: marketed tablet.

F1: optimized LSC formulation (refer Table 1 for composition).
